# ﻿Introducing *Melanocucurbitariauktampratovii* sp. nov. and the sexual morph of *Melanocamarosporioidesugamica* in Melanommataceae (Dothideomycetes, Pleosporales)

**DOI:** 10.3897/mycokeys.115.139963

**Published:** 2025-03-07

**Authors:** Dhandevi Pem, Rajesh Jeewon, Yusufjon Gafforov, Islomjon Urinboev, Abdulwahed Fahad Alrefaei, Indunil C. Senanayake, Alijon Esankulov, Kevin David Hyde

**Affiliations:** 1 School of Science, Mae Fah Luang University, Chiang Rai 57100, Thailand; 2 Center of Excellence in Fungal Research, Mae Fah Luang University, Chiang Rai 57100, Thailand; 3 Faculty of Medicine and Health Sciences, University of Mauritius, Moka, Mauritius; 4 Department of Zoology, College of Science, King Saud University, P.O. Box 2455, Riyadh 11451, Saudi Arabia; 5 Central Asian Center of Development Studies, New Uzbekistan University, Tashkent, 100007, Uzbekistan; 6 Department of Ecological Monitoring, National University of Uzbekistan, Tashkent, 100174, Uzbekistan; 7 Germplasm Bank of Wild Species & Yunnan Key Laboratory for Fungal Diversity and Green Development, Kunming Institute of Botany, Chinese Academy of Sciences, Kunming, 650201, China; 8 Scientific-Research Institute of Horticulture, Viticulture and Winemaking named after Academician M. Mirzaev, Tashkent, Uzbekistan; 9 Department of Botany and Microbiology, College of Science, King Saud University, P.O. Box 22452, Riyadh 11495, Saudi Arabia

**Keywords:** 1 new species, Central Asia, DNA, host, morphology, phylogeny, record, sequence

## Abstract

Most species of Melanommataceae are saprobic on decayed parts of various plants in tropical and temperate terrestrial habitats. During a survey of microfungi associated with terrestrial plants in Uzbekistan, two melanommataceous taxa were collected from dead branches of *Rosaecae* (Rosaceae) and *Salviakarelinii* (Lamiaceae). This study introduces a new species, *Melanocucurbitariauktampratovii*, and provides a new host and sexual morph record for *Melanocamarosporioidesugamica*, based on morphological observations and multi-gene phylogenetic analyses of concatenated LSU, SSU, ITS, and TEF-1 sequence data. *Melanocucurbitariauktampratovii* is the second species described within this genus and differs from the type species of *Melanocucurbitaria* in having smaller ascomata, smaller asci, smaller ascospores, and a different number of septa. The sexual morph of *Melanocamarosporioidesugamica* is characterized by globose to ovoid ascomata, cylindrical-clavate asci, ellipsoidal, muriform ascospores with 5–7 transversely septate, and 5–11 vertical septa. Illustrations and descriptions are provided, along with ecological and morphological comparisons of similar species within their respective genera.

## ﻿Introduction

Over the past 20 years, extensive research on members of Melanommataceae has yielded numerous taxonomic findings (36 genera, 337 species), largely attributed to advancements in DNA sequencing technology (Pem et al. 2019b; Hongsanan et al. 2020; Gao et al. 2023; Hyde et al. 2024a; Tennakoon et al. 2024). Species within Melanommataceae are classified based on morphological traits, such as the shape of fruiting bodies (conical or round), the type of peridium (cephalothecioid or non-cephalothecioid), the structure of pseudoparaphyses (cellular or trabeculate), the characteristics of asci (with short or furcate pedicels), and the nature of ascospores (monomorphic or dimorphic), along with the presence or absence of gelatinous sheaths, guttules, and germ pores (Wijayawardene et al. 2012; Almeida et al. 2017; Hyde et al. 2018; Dong et al. 2020). However, identifying species based solely on morphology can be challenging, as many species exhibit similar traits, leading to potential confusion. Consequently, modern fungal classification now commonly integrates both DNA-based phylogenetic analysis and morphological assessment coupled with consensus among mycologists for a better classification scheme (Hyde et al. 2023; Kularathnage et al. 2023; Tang et al. 2023; Dong et al. 2024; Sui et al. 2024; Tian et al. 2024).

The primary molecular markers employed in phylogenetic analyses of Melanommataceae (Pleosporales) include the 28S large subunit (LSU), 18S small subunit (SSU), internal transcribed spacers (ITS1–5.8S–ITS2), translation elongation factor 1 gene (TEF-1), and RNA polymerase second largest subunit (rpb2) (Tennakoon et al. 2018; Pem et al. 2019b). Melanommataceae is one of the highly diverse families within Dothideomycetes (Tian et al. 2015; Hongsanan et al. 2020; Pem et al. 2024). The family was introduced by Winter (1885), with *Melanomma* designated as the type genus for species characterized by globose to subglobose ascomata, clavate to nearly cylindrical asci, and fusoid to ellipsoidal or muriform ascospores. Members of Melanommataceae, which belong to Pleosporales, are found in various ecosystems and are known to thrive on a wide range of hosts globally (Gafforov 2017; Li et al. 2017; Pem et al. 2019b; Kularathnage et al. 2023). The cosmopolitan nature of Melanommataceae is underscored by the numerous new genera and species discovered in recent years (Li et al. 2016; Tennakoon et al. 2024). Despite being considered polyphyletic, the taxonomy of Melanommataceae remains unclear, as several genera lack sequence data (Zhang et al. 2012). Several researchers have advocated the inclusion of DNA sequence data with additional sample collections to genera that have insufficient data and for clarifying the confusion between sexual and asexual morphs to stabilize taxonomy (Shenoy et al. 2007; Shenoy et al. 2010; Karunarathna et al. 2017; Hongsanan et al. 2020). To date, there are 26 genera exhibiting sexual morphs and 10 genera exhibiting asexual morphs within Melanommataceae (Hongsanan et al. 2020; Pem et al. 2024). Gao et al. (2023) identified a pleomorphic genus, *Dematiomelanomma*, from grassland vegetation in Yunnan, China, emphasizing the importance of pleomorphism in Melanommataceae. Tennakoon et al. (2024) highlighted the host associations and geographical distribution of *Melanommataceous* species.

Most Melanommataceae species are reported from Western regions, including Europe and North America (Tennakoon et al. 2024). Targeting underexplored regions such as Central Asia, including Uzbekistan, might be helpful for the discovery of new fungi(Gafforov 2017; Kan et al. 2017; Hyde et al. 2019; Cheek et al. 2020; Hyde et al. 2024b; Gafforov et al. 2025). Recent studies have led to the discovery of several new genera and species of ascomycetes in arid regions, particularly from Uzbekistan, Central Asia (Gafforov and Hoshino 2015; Gafforov and Rakhimov 2017; Pem et al. 2018, 2019a, 2019b; Gafforov et al. 2019; Abdurazakov et al. 2021; Appadoo et al. 2021; Htet et al. 2021; Lestari et al. 2021; Aluthmuhandiram et al. 2022; Dong et al. 2023; Senwanna et al. 2024). *Melanocamarosporoides* and *Melanocucurbitaria* were first discovered from Uzbekistan (Wanasinghe et al. 2018; Pem et al. 2019b). However, the two genera are still poorly known worldwide. The aim of the present study was to clarify the taxonomic position of a new species and new record of *Melanocucurbitaria* and *Melanocamarosporioides* and to identify new taxa through multi-gene phylogeny and morphological examination.

## ﻿Materials and methods

### ﻿Sample collection, morphological examination and isolation

Fresh fungal specimens were collected from dead trunks and branches in the Surkhandarya and Tashkent provinces of Uzbekistan, and the important collection information was noted (Rathnayaka et al. 2024). The samples were transported to the laboratory in zip-lock plastic bags and incubated for 24 hours in plastic containers lined with wet tissue paper. The micromorphological characters were examined following the methods described by Pem et al. (2019b). Vertical sections of the ascomata were made using a razor blade and mounted in distilled water on a glass slide. A stereomicroscope (Motic series SMZ-171) was used to observe the surface morphology of fungal fruiting bodies. Micro-morphological structures were examined under a Nikon Eclipse 80i compound microscope, and photographs were captured with a Canon 600D digital camera fitted to the compound microscope using differential interference contrast (DIC) microscopy. The micro-morphological features, including shape, structure, and color, were meticulously recorded. Asci were stained with Melzer’s reagent to assess the reactions of the apical ring, while ascospores were stained with Indian ink to evaluate the presence of gelatinous sheaths surrounding them. All measurements, including the height and width of ascomata, asci, ascospores, peridium, and pseudoparaphyses, were made using the Tarosoft® Image Framework program. Photo plates were created using Adobe Photoshop CS3 Extended Version 10.0 (Adobe Systems, USA). Single spore isolation was conducted following the method described by Senanayake et al. (2020). Germinated spores were transferred to malt extract agar (MEA) plates and incubated at 16 °C under daylight, as outlined by Liu et al. (2010). Colony color and other characters were observed, and growth rates were measured after one week and at subsequent weekly intervals. The holotype specimen was deposited in the Tashkent Mycological Herbarium (TASM) at the Institute of Botany, Academy of Sciences of the Republic of Uzbekistan, while an isotype specimen was deposited in the Mae Fah Luang University Herbarium (MFLU) in Chiang Rai, Thailand. Living cultures were submitted to the Culture Collection at Mae Fah Luang University (MFLUCC). The Faces of Fungi (FoF) and Index Fungorum (IF) numbers were provided as outlined by Jayasiri et al. (2015) and Index Fungorum (2024). New taxon and species identification were based on the recommendations of Jeewon and Hyde (2016), Aime et al. (2021), Chethana et al. (2021), and Pem et al. (2021).

### ﻿DNA extraction, PCR amplification, and sequencing

Genomic DNA was extracted from fresh fungal mycelium using a DNA extraction kit (E.Z.N.A Fungal DNA Mini Kit, D3390-02, Omega Bio-Tek) following the manufacturer’s protocol. The DNA product was kept at 4 °C for DNA amplification, and duplicates were maintained at -20 °C for long-term storage. The primers LR0R/LR5 were used to amplify the 28S large subunit rDNA (LSU) (Vilgalys and Hester 1990), NS1/NS4 for 18S small subunit ribosomal RNA (SSU) (White et al. 1990), ITS4/ITS5 for the 5.8S nrRNA gene with the two flanking internal transcribed spacers (ITS) (White et al. 1990), and EF1-983F/EF1-2218R primers for the partial translation elongation factor 1-alpha (TEF-1) (Rehner 2001).The amplification reactions were achieved in a total reaction volume of 25 µl, which comprised 9.5 µ l of sterilized distilled water, 12.5 µl of 2 × Power Taq PCR MasterMix (a ready- to-use mixture, including DNA polymerase, the NH_4_^+^ buffer system, dNTPs, magnesium chloride, and an inactive red dye and stabilizer) (Bioteke Co., China), 1 μl of each forward and reverse primer and 1 μl of DNA template. The polymerase chain reaction (PCR) thermal cycle program for LSU, SSU, ITS and TEF-1 gene regions was followed as detailed by Pem et al. (2019b). The quality of the PCR products was checked with 1% agarose gel electrophoresis containing the SafeView^TM^. The purified PCR products were sequenced at Sangon Biotech (Shanghai) Co., Ltd., China. Generated nucleotide sequence data were deposited in GenBank, and accession numbers were recorded (Table 1).

**Table 1.** Culture collection code and GenBank accession numbers of fungal strains used for phylogenetic analysis in this study.“*” Denotes ex-type, ex-isotype, ex-paratype or ex-epitype strains. “^T^” Denotes type species. Newly generated sequences are displayed in bold. NA: sequence data is not available.

Taxon	Strain/ Culture accession no	GenBank accession No.			
		ITS	LSU	SSU	TEF-1
*Alpinariarhododendri* ^T^	KT 2520	LC203335	LC203360	LC203314	LC203388
*Alpinariarhododendri* ^T^	MFLU 20-0278	MT229210	MT229208	MT229209	MT254066
*Aposphaeriacorallinolutea*	MFLU 15-2752	KY554202	KY554197	KY554200	KY554205
*Aposphaeriacorallinolutea*	MFLU 16-2412	MT177916	MT177943	MT177971	NA
*Bertiellaellipsoidea*	MFLUCC 17-2015	MG543922	MG543913	NA	MG547226
*Bertiellafici*	NCYU 19-0073*	NA	MW063224	MW079352	MW183787
*Beverwykellapulmonaria* ^T^	CBS 283.53*	MH857201	MH868739	NG_061258	NA
*Byssosphaeriamacarangae*	MFLUCC 17-2655*	MH389782	MH389778	MH389780	MH389784
*Byssosphaeriataiwanense*	MFLUCC 17-2643*	MH389783	MH389779	MH389781	MH389785
*Camposporiumdulciaquae*	MFLU 21-0015*	MT864352	MT860430	MW485612	MW537104
*Camposporiumseptatum*	MFLUCC 19-0483*	MN758892	MN759023	MN758958	MN784096
*Cyclothyriellarubronotata* ^T^	CBS 121892	KX650541	NA	NA	KX650516
*Cyclothyriellarubronotata* ^T^	CBS 385.39	MH856047	JX681121	AY642521	NA
*Dematiomelanommayunnanense* ^T^	KUNCC 23-12728*	OQ225528	OQ360647	OQ360651	OQ413238
*Dematiomelanommayunnanense* ^T^	KUNCC 23-12730	OQ225529	OQ360648	OQ360652	OQ413239
*Dematiomelanommayunnanense* ^T^	CGMCC 3.23744	OQ225530	OQ360649	OQ360653	OQ413240
*Dematiomelanommayunnanense* ^T^	KUNCC 22-12677	OQ225531	OQ360650	OQ360654	OQ413241
*Fusiconidiummackenziei* ^T^	MFLUCC 14-0434*	NA	KX611112	KX611114	KX611118
*Gemmamycespiceae*	C251	KY189977	NA	NA	KY190012
*Gemmamycespiceae*	C209	KY189976	NA	KY190006	KY190011
*Herpotrichiajuniperi*	CBS 200.31	NA	DQ678080	DQ678029	DQ677925
*Herpotrichiamacrotricha*	GKM 196N	NA	GU385176	NA	GU327755
*Herpotrichiaxiaokongense*	KUMCC 21-0004*	NA	MZ408889	MZ408891	MZ394066
*Marjiatianshanica* ^T^	TASM 6121*	MG828910	MG829020	MG829127	MG829207
*Marjiauzbekistanica*	TASM 6122*	MG828911	MG829021	MG829128	MG829208
*Melanocamarosporiumgaliicola* ^T^	MFLUCC 13-0545*	NA	OR206417	OR206407	NA
*Melanocamarosporioidesugamica* ^T^	MFLU 17-0064*	MH000192	MH000190	MH000191	MH006610
***Melanocamarosporioidesugamica* ^T^**	**TASM 6175**	**PQ453019**	**PQ453821**	**PQ433587**	
*Melanocucurbitariauzbekistanica* ^T^	MFLUCC 17-0829*	MG828912	MG829022	MG829129	MG829209
***Melanocucurbitariauktampratovii* ^T^**	**TASM 6176**	**PQ453018**	**PQ453820**		**PQ441826**
*Melanodiplodiatianschanica* ^T^	MFLUCC 17-0805*	MG828913	MG829023	MG829130	MG829210
*Melanodiplodiatianschanica* ^T^	TASM 6111*	MG828914	MG829024	MG829131	MG829211
*Melanodiplodiatianschanica* ^T^	TASM 6112	MG828915	MG829025	MG829132	MG829212
*Melanommajaponicum*	MAFF 239634*	LC203321	LC203339	LC203293	LC203367
*Melanommajaponicum*	KT 3425*	LC203320	LC203338	LC203292	LC203366
*Melanommapulvis-pyrius* ^T^	CBS 124080*	MH863349	GU456323	GU456302	GU456265
*Monoseptellarosae* ^T^	MFLUCC 17-0815*	MG828916	MG829026	MG829133	MG829213
*Muriformistrickeriarosae* ^T^	MFLU 16-0227*	MG828918	MG829028	MG829135	MG829215
*Muriformistrickeriarubi* ^T^	MFLUCC 17-2550	MG828919	MG829029	MG829136	MG829216
*Muriformistrickeriarubi* ^T^	MFLUCC 15-0681*	NA	KT934253	KT934257	KT934261
*Neobyssosphaeriaclematidis* ^T^	MFLUCC 17-0794*	NA	MT214566	MT408594	NA
*Petrakiaechinata* ^T^	L54	NA	NA	KY190007	KY190015
*Petrakiaechinata* ^T^	CBS 133070	JQ691628	LC203352	LC203306	LC203380
*Phragmocephalaatra*	MFLUCC 15-0021	KP698721	KP698725	KP698729	NA
*Phragmotrichumchailletii* ^T^	CPC 33263*	MN313812	MN317293	NA	MN313858
*Phragmotrichumchailletii* ^T^	CPC 33341	MN313813	MN317294	NA	MN313859
*Phragmocephalagarethjonesii*	MFLUCC 15-0018*	KP698722	KP698726	KP698730	NA
*Pleotrichocladiumopacum* ^T^	AU-BD04	JN995638	JN941370	JN938733	NA
*Pleotrichocladiumopacum* ^T^	FMR 12416*	KY853462	KY853523	NA	NA
*Praetumpfiaobducens* ^T^	C2	KY189982	NA	NA	KY190017
*Praetumpfía obducens* ^T^	C54	KY189984	NA	KY190008	KY190019
*Pseudobyssosphaeriabambusae* ^T^	MFLU 18-0151*	MG737556	MG737555	NA	MG737557
*Pseudodidymellaminima*	KT 2918*	LC203333	LC203358	LC203312	LC203386
*Pseudodidymellafagi* ^T^	H 2579*	LC150787	LC203356	LC203310	LC203384
*Pseudostrickeriaononidis*	MFLUCC 14-0949*	NA	KT934255	KT934259	KT934263
*Pseudostrickeriarosae*	MFLUCC 17-0643*	MG828954	MG829065	MG829169	MG829234
*Pseudotrichiamutabilis*	SMH 1541	NA	GU385209	NA	NA
*Pseudotrichiamutabilis*	WU 36923	KY189988	NA	NA	KY190022
*Sarimanaspseudofluviatile*	KT760*	LC001717	LC001714	LC001711	NA
*Sarimanasshirakamiense* ^T^	HHUF 30454*	NR_138017	NG_059803	NG_061263	NA
*Seifertiaalpina*	ZT Myc 59953*	MK502003	MK502026	MK502037	MK502083
*Seifertiaazaleae* ^T^	ZT Myc 59954	MK502004	MK502028	MK502038	MK502085
*Tumulariaaquatica*	CBS 212.46*	MH856165	MH867689	NA	NA
*Tumulariatuberculata* ^T^	CBS 256.84	NA	GU301851	NA	GU349006
*Uzbekistanicarosae-hissaricae* ^T^	MFLUCC 17-0819*	MG828975	MG829087	MG829187	MG829242
*Uzbekistanicayakutkhanika* ^T^	MFLUCC 17-0842*	MG828978	MG829090	MG829190	MG829245
*Uzbekistanicapruni* ^T^	MFLU 17-2136*	MN758893	MN759024	NA	MN784097
*Uzbekistanicavitis-viniferae* ^T^	CPC 35793*	MT223867	MT223938	NA	NA
*Xenostigminazilleri* ^T^	CBS 115685	FJ839638	FJ839674	LC203316	LC203390
*Xenostigminazilleri* ^T^	CBS 115686	GU269841	FJ839676	LC203317	LC203391

### ﻿Sequence alignment and phylogenetic analyses

The contigs (forward and reverse sequences) were merged using SeqMan (version 7.0.0; DNASTAR, Madison, WI, USA). Assembled sequences were put through a BLAST search in GenBank to find highly similar strains (https://blast.ncbi.nlm.nih.gov/). The other sequences used in the analyses were obtained from recent publications (Gao et al. 2023, Tennakoon et al. 2024). The combined dataset comprised 68 isolates, including *Cyclothyriellarubronotata* (CBS 121892, CBS 141486) as the outgroup taxa. Single gene sequences were aligned using the online MAFFT v.7.526 program (https://mafft.cbrc.jp/alignment/software/) (Katoh and Standley 2013) and improved manually where necessary. Single-gene alignments were combined using BioEdit v.7.2.5 (Hall 1999). Maximum Likelihood (ML) and Bayesian Inference (BI) were performed to analyze the concatenated aligned dataset. Maximum Likelihood analysis was performed using the CIPRES Science Gateway v. 3.3 online platform, with RAxML-HPC v.8 on XSEDE (8.2.12) software, employing the GTR+I+G nucleotide evolution model (Stamatakis et al. 2008; Miller et al. 2010; Stamatakis 2014). The best-fit nucleotide substitution models for individual barcodes were determined with MrModelTest v. 2.3 (Nylander 2004) based on the Akaike Information Criterion (AIC). The Bayesian Inference phylogeny was carried out using MrBayes 3.2.1 (Ronquist et al. 2012), with four chains of 2,000,000 generations and sampling trees every 100^th^ generation. The first 20% of the sampled data was removed as burn-in. The phylograms were viewed with the FigTree v.1.4.0 program (Rambaut et al. 2018) and rearranged in Adobe Illustrator® CS3 (Version 15.0.0, Adobe®, San Jose, CA). The alignments and sequences were submitted to TreeBASE (http://www.treebase.org/) and GenBank (https://www.ncbi.nlm.nih.gov/).

## ﻿Results

### ﻿Phylogenetic analyses

**Figure 1.**
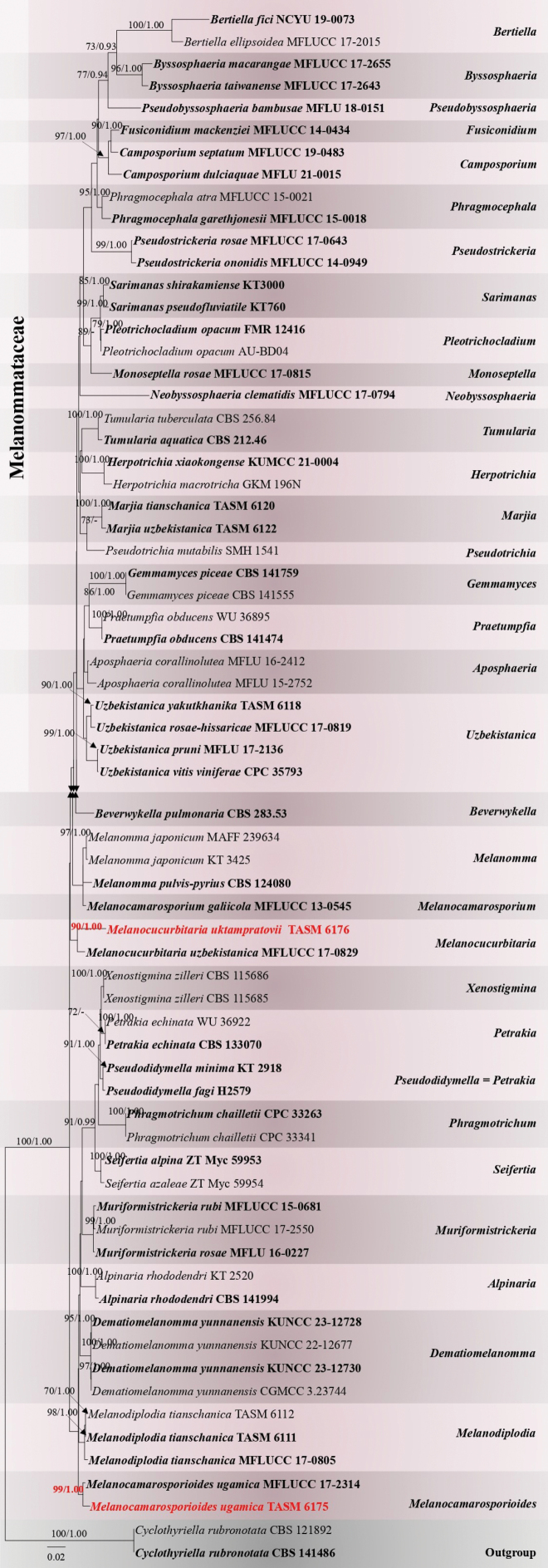
Phylogram generated from maximum likelihood analysis based on combined LSU, SSU, ITS and TEF-1 sequence data for Melanommataceae. The tree is rooted with *Cyclothyriellarubronotata* (CBS 121892, CBS 141486). The new isolates are in red and ex-type strains and are shown in boldface. Bootstrap support values for maximum likelihood (ML) equal to or greater than 70% and Bayesian posterior probabilities (BYPP) equal to or greater than 0.90 are given above the nodes, respectively.

Single and multi-gene analyses of LSU, SSU, ITS, and TEF-1 were conducted on all accessible sequences of Melanommataceae species to compare tree topology and clade stability (data not shown). Based on these analyses and BLAST results, 68 isolates, including outgroup taxa, were selected for the combined gene analysis (Table 1). The phylogenetic analyses incorporated 3,030 characters, including gaps, from the combined LSU, SSU, ITS, and TEF-1 sequences. The RAxML analysis of the combined data set generated the best scoring tree (Fig. 1). The final ML optimization likelihood value was -14235.344070. There were 22.81% undetermined characters or gaps and 853 distinct alignment patterns. Estimated base frequencies were A = 0.249838, C = 0.234863, G = 0.267108, T = 0.248191; substitution rates AC = 2.297893, AG = 3.198741, AT = 1.875479, CG = 1.160970, CT = 13.099544, GT = 1.0; proportion of invariable sites I = 0.612009; gamma distribution shape parameter α = 0.532932. The Bayesian analysis has resulted in 20,000 trees after 2,000,000 generations. All analyses (ML and BYPP) showed similar topologies and agreed with previous studies (Gao et al. 2023; Tennakoon et al. 2024). According to the multi-gene phylogeny, TASM 6176 groups in a sister clade to *Melanocucurbitariauzbekistanica* (MFLUCC 17-0829) with 90% ML and 1.00 BYPP statistical support. Our isolate MFLUCC 24-0466 clusters with *Melanocamarosporioidesugamica* (MFLUCC 17-2314) with 99% ML and 1.00 BYPP statistical support.

### ﻿Taxonomy

#### ﻿ Melanocucurbitaria uktampratovii

Taxon classificationFungiPleosporalesMelanommataceae

D. Pem, R. Jeewon, Gafforov & K. D. Hyde sp. nov.

EBDBEF5A-DDFE-5F31-A35C-8BEDD9F03C16

Index Fungorum: IF902644

Facesoffungi Number: FoF16743

Fig. 2

##### Etymology.

uktam-pratovii (Lat.) in honor of Uzbek scientist, Prof. Uktam Pratovich Pratov (1934–2018), for his contribution to the botanical research in Central Asian countries.

**Figure 2.**
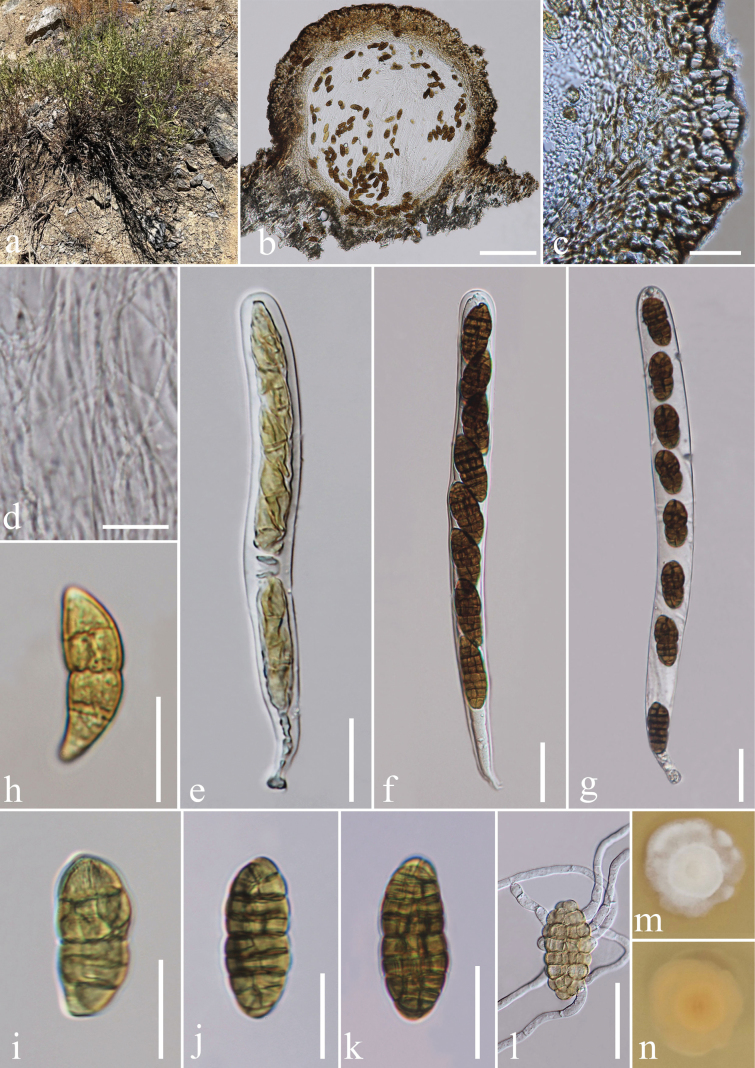
*Melanocucurbitariauktampratovii* sp. nov. (TASM 6176, holotype) **a** habitat **b** section of ascoma **c** peridium **d** pseudoparaphyses **e–g** asci **h–k** ascospores **l** germinated ascospore **m, n** culture characteristics on MEA (**m** above view, **n** reverse view). Scale bars: 100 μm (**b**); 20 μm (**c, e–l**); 10 μm (**d**).

##### Description.

***Saprobic*** on dead branches of *Salviakarelinii* J. B. Walker. ***Sexual morph*: *Ascomata*** 200–500 μm wide, 250–400 μm high scattered to gregarious, immersed or semi erumpent, carbonaceous, dark brown to black, globose to subglobose, papillate. ***Ostiole*** indistinct, with a small papilla. ***Peridium*** 25–35 μm, 3–5 layers, inner layers composed of subhyaline to light brown cells of *textura angularis*, outer layer composed of irregular, thick-walled, highly pigmented dark-brown cells of *textura angularis*. ***Hamathecium*** 1.5–2.0 µm (n = 10) wide, comprising numerous, filamentous, branched, anastomosing, septate, hyaline, pseudoparaphyses. ***Asci*** 160–175 × 13–14 μm (x̄ = 164.0 × 13.6 µm, n = 10), 8-spored, bitunicate, fissitunicate, cylindrical, pedicellate, apically rounded, with an ocular chamber. ***Ascospores*** 18–25 × 7–9 μm (x̄ = 20.8 × 7.7 µm, n = 10), uniseriate, ellipsoidal, muriform, 4–6 transverse septa, with 3–7 longitudinal septa, slightly constricted at the septa, more at the middle septum, at first hyaline turning golden brown to dark-brown at maturity, obtuse at the ends, lacking a mucilaginous sheath, smooth and thick-walled. ***Asexual morph***: Undetermined.

##### Culture characteristics.

Colonies on MEA, reaching 20–25 mm diam. after 3 weeks at 25 °C, medium dense in middle, medium sparse at the edges, irregular, umbonate, velvety to floccose, undulated edges, smooth.

##### Known distribution

**(based on molecular data).** Uzbekistan (Wanasinghe et al. 2018; this study).

##### Confirmed hosts

**(based on molecular data).***Acerpubescens* Franch (Sapindaceae) (Wanasinghe et al. 2018), *Salviakarelinii* (Lamiaceae) (this study).

##### Material examined.

Uzbekistan • Surkhandarya Province, Baysun District, Omonxona Village, South-Western Hissar Mountains, on dead branches of *Salviakarelinii* (Lamiaceae), 13 May 2016, Y. Gafforov, I. Urinboev, YG-S29-3 (TASM 6176, holotype; MFLU 17-0071, isotype), ex-type living culture MFLUCC 17-1953.

##### GenBank numbers.

LSU: PQ453820, ITS: PQ453018, TEF-1: PQ441826.

##### Notes.

The morphology of our collection (TASM 6176) resembles the type species *Melanocucurbitariauzbekistanica* (TASM 6109) in its scattered to gregarious, dark brown to black, globose to subglobose, papillate ascomata, cylindrical, pedicellate asci and ellipsoidal, muriform ascospores (Wanasinghe et al. 2018). Our collection is different from the type species *M.uzbekistanica* in having smaller ascomata (200–400 × 250–400 μm vs. 500–700 × 550–750 μm), smaller asci (160–175 × 13–14 μm vs. 280–300 × 19–23 µm), smaller ascospores (18–25 × 7–9 μm vs. 37–47 × 17–19 µm) and number of septa (4–6 transverse septa, 3–7 longitudinal septa vs. 6–8 transverse septa, 3–4 longitudinal septa) (Wanasinghe et al. 2018). *Melanocucurbitariauzbekistanica* also differs from *M.uktampratovii* in having black, semi-immersed ascomata, ostiole filled with brown cells, ascospores which are pale brown at maturity, broadly rounded at the ends, surrounded by a mucilaginous sheath when immature while *M.uktampratovii* has immersed or semi erumpent ascomata, indistinct ostiole, with a small papilla, ascospores which are dark brown at maturity, obtuse at the ends and lacking a mucilaginous sheath. According to the multi-gene phylogeny, our collection clusters with the isolate of *Melanocucurbitariauzbekistanica* (MFLUCC 17-0829) in 90% ML and 1.00 BYPP supported clade. The nucleotide base comparison of LSU, ITS and TEF-1 regions showed that our strain (TASM 6176) differs from the type strain of *M.uzbekistanica* (MFLUCC 17-0829) by 23/865 bp (2.65%), 15/439 bp (3.41%) and 20/571 bp (3.5%), respectively. Therefore, we introduce our collection as a new species based on morphology and phylogeny. The phylogenetic placement of our strain (TASM 6176) is shown in Fig. 1.

#### ﻿ Melanocamarosporioides ugamica

Taxon classificationFungiPleosporalesMelanommataceae

D. Pem, R. Jeewon, Gafforov & K. D. Hyde, in Pem et al. Mycol. Progr. 18(3): 474 (2019)

4474C9BD-48C4-5263-8AB9-A09C0EC19C8F

Index Fungorum: IF554297

Facesoffungi Number: FoF04363

Fig. 3

##### Description.

***Saprobic*** on dead branches of *Rosaecae* Aitch. ***Sexual morph*: *Ascomata*** 145–335 μm wide, 70–195 μm high, solitary to gregarious, flattened, semi-immersed to superficial, dark brown to black, globose to ovoid, carbonaceous, papillate. ***Peridium*** 15–20 μm, two layered, inner layers composed of hyaline to subhyaline cells of *textura prismatica*, outer layer composed of irregular, thick-walled, brown cells of *textura angularis*. ***Hamathecium*** comprising numerous, 1.3–2.5 µm (n = 10) wide, filamentous, branched, anastomosing, septate, hyaline, pseudoparaphyses. ***Asci*** 90–120 × 14.5–16.7 μm (x̄ = 104.9 × 15.5 µm, n = 10), 8-spored, bitunicate, fissitunicate, cylindrical-clavate, pedicellate, apically rounded, with a minute ocular chamber. ***Ascospores*** 19.2–25.0 × 7.9–10.0 μm (x̄ = 21.8 × 8.7 µm, n = 10), uni to biseriate, ellipsoidal, muriform, 5–7 transversely septate, with 5–11 vertical septa, slightly constricted at the septa, dark brown, broadly rounded at the ends, smooth and thick-walled. ***Asexual morph***: Undetermined.

**Figure 3.**
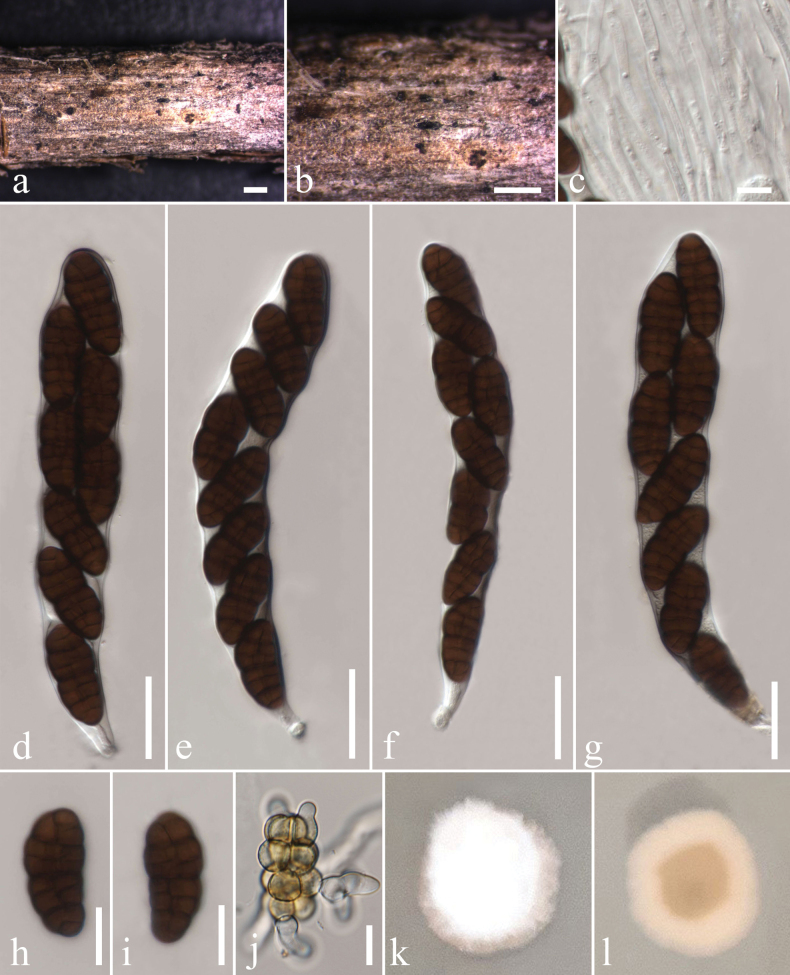
*Melanocamarosporioidesugamica* (TASM 6175, new host record and sexual morph) **a, b** appearance of ascomata on the host surface **c** pseudoparaphyses **d–g** asci **h, i** ascospores **j** germinated ascospore **k, l** culture characteristics on MEA (**k** above view, **l** reverse view). Scale bars: 500 μm (**a, b**); 5 μm (**c**); 20 μm (**d–g, j**); 10 μm (**h, i**).

##### Culture characteristics.

Colonies on MEA, reaching 25–30 mm diam. after 3 weeks at 25 °C, medium dense, circular, flattened to slightly raised, smooth surface, with edge entire, cottony, mycelium composed of septate, branched hyphae, colony from above whitish, reverse whitish gray to reddish brown in center gradually becoming white towards the edges from the below.

##### Known distribution

**(based on molecular data).** Uzbekistan (Pem et al. 2019b, this study).

##### Confirmed hosts

**(based on molecular data).***Loniceraaltmannii* (Caprifoliaceae) (Pem et al. 2019b), *Rosaecae* (this study).

##### Material examined.

Uzbekistan • Tashkent Province, Bostanlik District, Ugam-Chatkal National Nature Park, Charvak Reservoir, Chimyon in Western Tien Shan Mountain, on dead branches of *Rosaecae*, 21 July 2019, Y. Gafforov, A. Esankulov, YG-S2-2 (TASM 6175), living culture MFLUCC 24-0466.

##### GenBank numbers.

LSU: PQ453821, SSU: PQ433587, ITS: PQ453019.

##### Notes.

Our new isolate MFLUCC 24-0466 is morphologically and phylogenetically related to *Melanocamarosporioidesugamica* (MFLUCC 17-2314) but collected from a different host. *Melanocamarosporioidesugamica* was reported from Uzbekistan on dead trunk and branches of *Loniceraaltmannii* (Caprifoliaceae) (Pem et al. 2019b), while our collection was found on dead branches of *Rosaecae*. *Melanocamarosporioidesugamica* (TASM 6133) is characterized by an asexual morph, namely black, globose, superficial conidiomata and large conidia, which are multiseptate and distinctively dark brown, while our collection (TASM 6175) is in its sexual state, characterized by globose to ovoid, dark brown to black ascomata, cylindrical asci, and dark brown, ellipsoidal, muriform ascospores. Multi-gene phylogeny (LSU, SSU, ITS, and TEF-1) shows that our collection groups with *Melanocamarosporioidesugamica* (MFLUCC 17-2314) in a 99% ML and 1.00 BYPP supported clade. With regard to DNA sequence data comparison, there is a difference of 0.69% (6 out of 865), 0.84% (9 out of 1067), and 0% (0 out of 470) in nucleotide variations within the LSU, SSU, and ITS genes, respectively. Hence, we introduce our collection as a new host record of *Melanocamarosporioides* from *Rosaecae*. This is also the first sexual morph report in *Melanocamarosporioides*. The phylogenetic placement of our strain TASM 6175 is shown in Fig. 1.

## ﻿Discussion

During surveys on saprobic fungiassociated with flowering plants in Uzbekistan, we found two taxa, which belong to Melanommataceae. The newly described taxon is *Melanocucurbitariauktampratovii*, which exhibits distinct characteristics compared to the type species *M.uzbekistanica*. *Melanocucurbitariauktampratovii* is characterized by scattered to gregarious, globose to subglobose, dark brown to black ascomata; cylindrical, pedicellate asci with an ocular chamber; ellipsoidal, muriform ascospores with 4–6 transverse septa and 3–7 longitudinal septa. *Melanocucurbitariauzbekistanica* is characterized by larger ascomata, asci, and ascospores with 6–8 transverse septa and 3–4 longitudinal septa compared to *M.uktampratovii*, which has 4–6 transverse septa and 3–7 longitudinal septa. In our phylogeny, *Melanocucurbitariauktampratovii* is closely related to *M.uzbekistanica. Melanocucurbitariauzbekistanica* is characterized by larger ascomata, asci, and ascospores compared to *M.uktampratovii*. *Melanocucurbitariauktampratovii* bears morphological resemblance to the type species of *Gemmamyces*, *Melanocamarosporoides*, *Muriformistrickeria*, *Praetumpfia*, *Pseudostrickeria*, and *Uzbekistanica* in having muriform ascospores (Wanasinghe et al. 2018; Gao et al. 2023), but *M.uktampratovii* is distinctly separated from these genera in phylogenetic analysis. In addition, a comparison of nucleotides across several genes also supports that our new taxon is sufficiently distinct to warrant its establishment as a new species.

*Melanocamarosporoidesugamica* is the type species of *Melanocamarosporoides*. Pem et al. (2019b) introduced the asexual morph of *Melanocamarosporioides* collected on dead trunks and branches of *Loniceraaltmannii* (Caprifoliaceae) in Uzbekistan. *Melanocamarosporioides* is distinct from other genera in Melanommataceae based on its multiseptate, large, dark-brown conidia (Pem et al. 2019b). Up to now, the sexual state has not been observed. In this study, we found the sexual morph of *Melanocamarosporoidesugamica* isolated for the first time from dead branches of *Rosaecae* in Uzbekistan. So far, there is only one species in this genus, and its relationships were not well resolved in previous studies. In our study, multigene phylogeny shows a strong relationship with *Melanodiplodiatianschanica* and *Dematiomelanommayunnanense*, but *Melanocamarosporioidesugamica* (TASM 6175) can be differentiated based on various morphological features. *Melanodiplodia* produces diplodia-like conidia (Wanasinghe et al. 2018), *Dematiomelanomma* has camarographium-like conidia, whereas *Melanocamarosporioides* forms camarosporium-like conidia (Pem et al. 2019b). Furthermore, the sexual morph of *Melanocamarosporioidesugamica* and *Dematiomelanommayunnanense* are different in their asci (cylindrical vs. cylindrical-clavate) and ascospore (5–7 transversely septate, with 5–11 vertical septa, lacking a sheath vs. 3–7 transversely septate, and 1–3 vertical septa with a mucilaginous sheath) characteristics. Further sampling is necessary to improve our knowledge of the diversity and ecology of Melanommataceae species on flowering plants in arid and semi-arid habitats.

## ﻿Conclusion

This study describes a new species in *Melanocucurbitaria* and provides a new record of the sexual morph in *Melanocamarosporioides*, using both morphological and molecular data. To date, there are 25 sexual and 10 asexual morph genera within Melanommataceae (Hongsanan et al. 2020; Pem et al. 2024). In addition to the description of new species, it is essential to elucidate the relationships between sexual and asexual morphs to fully understand the life cycles of microfungi and enhance fungal taxonomy (Lücking et al. 2020; Zhou and May 2022). This understanding will aid in accurately estimating the total number of fungal species worldwide, as previous global estimates relied heavily on the ratio of fungito their occurrences on hosts (Wu et al. 2019; Niskanen et al. 2023). Numerous ascomycete and basidiomycetous fungihave been discovered in Uzbekistan, and it is likely that many more species await identification in this region. Therefore, conducting comprehensive mycological research in this Central Asia region is essential to uncover its full fungal diversity.

## Supplementary Material

XML Treatment for Melanocucurbitaria uktampratovii

XML Treatment for Melanocamarosporioides ugamica

## ﻿Additional information

### Author ORCIDs

Dhandevi Pem https://orcid.org/0000-0003-1831-9266

Rajesh Jeewon https://orcid.org/0000-0002-8563-957X

Yusufjon Gafforov https://orcid.org/0000-0003-3076-4709

Islomjon Urinboev https://orcid.org/0009-0009-1653-000X

Abdulwahed Fahad Alrefaei https://orcid.org/0000-0002-3761-6656

Indunil C. Senanayake https://orcid.org/0000-0002-7165-2394

Alijon Esankulov https://orcid.org/0009-0000-1862-7555

Kevin David Hyde https://orcid.org/0000-0002-2191-0762

### Data availability

All of the data that support the findings of this study are available in the main text.
